# Cardiac catherization in Austria: Results from the Austrian National Cardiac Catheterization Laboratory Registry (ANCALAR) 2012–2018

**DOI:** 10.1007/s00508-019-01599-4

**Published:** 2020-01-29

**Authors:** Volker Mühlberger, Lalit Kaltenbach, Katie Bates, Hanno Ulmer, Austrian National Cardiac Catheterization Laboratory Registry (ANCALAR), Österreichische Kardiologische Gesellschaft (ÖKG)

**Affiliations:** 1 Ordination Professor Mühlberger, Innrain 46, 6020 Innsbruck, Austria; 2 Department of Medical Statistics, Informatics und Health Economics, Medical University of Innsbruck, Schöpfstr. 41/1, 6020 Innsbruck, Austria

**Keywords:** Percutaneous coronary intervention, Coronary angiography, Cardiology, Austria

## Abstract

**Background:**

Cardiac catheterization is one of the most widely performed cardiac interventional procedures worldwide. The Austrian National Catheterization Laboratory Registry (ANCALAR), started in 1992, collects annual data on cardiac catheterization in Austria. The registry enables in-depth understanding of the dynamics of cardiac catheterization procedures and their use across 34 cardiac catheterization laboratories in Austria.

**Methods:**

Data from ANCALAR on cardiac catheterization including the latest data for 2017, voluntarily provided by centers with cardiac catheterization laboratories, were analyzed. Where possible, international comparisons in therapeutic and interventional cardiac procedures are made with Switzerland and Germany.

**Results:**

Internationally, Austria ranks alongside the top countries in Europe. Whilst the number of people undergoing routine percutaneous coronary interventions (PCI) remains stable, complex and acute interventions are increasing year by year in Austria.

**Conclusion:**

Evidence from ANCALAR revealed that Austria is another example of the difficulties of weighing current guidelines with new emerging evidence and resulting real-life clinical practice in the dynamic world of interventional cardiology.

**Electronic supplementary material:**

The online version of this article (10.1007/s00508-019-01599-4) contains supplementary material, which is available to authorized users.

## Introduction

Cardiovascular diseases, in particular coronary artery disease (CAD), remain the world’s leading cause of mortality and morbidity [1]. The gold standard for diagnosis and intervention in CAD remains cardiac catheterization, angiography and percutaneous coronary intervention (PCI) [2]. Cardiac catheterization is one of the most widely performed cardiac interventional procedures worldwide, it is a high-cost, high-risk procedure and its history has been characterized by rapid advances in both technique and technology [3]. For such a dynamic field of medicine, registries provide a means to monitor adherence to international guidelines, standards of care and enable benchmarking at the subnational and national level [4].

The Austrian National Catheterization Laboratory Registry (ANCALAR) is an observational registry that collects data on cardiac catheterization in Austria. Data have been collected annually since 1992, data are submitted on a voluntary basis by hospitals performing interventional procedures in Austria, the data are then centrally processed and analyzed. The ANCALAR is a valuable resource, revealing the everyday practice of interventional cardiology in Austria and enabling international comparisons.

With new data from 2017 now available, trends in cardiac catheterization in Austria from 2012–2017 are described, contextualised with reference to both international treatment guidelines and international comparisons with Germany and Switzerland.

## Data and methods

Data on diagnostic and interventional cardiac procedures in 2017 in the Austrian National Catheterization Laboratory Registry (ANCALAR) were used. In line with previous research, data were compared to the national cardiac catheterization registries of Germany and Switzerland [5–10].

ANCALAR is a voluntary, financially independent registry, maintained co-operatively by participating performing hospitals in Austria, coordinated by its initiator, Professor Mühlberger. The data are securely stored centrally and processed by the Department for Medical Statistics, Informatics and Health Economics at the Medical University Innsbruck. Whilst participation in the registry is voluntary, all hospitals providing interventional cardiac procedures in Austria participate, thus the data represent all cardiac intervention in Austria [5, 6].

Since 1992, the registry has collected over 90 parameters concerning cardiac catheterization, without interruption. Data are collected in accordance with the cardiology audit and registration data standards (CARDS) [11, 12]. Data collection tools are reviewed annually by the Interventional Cardiology working group of the Austrian Cardiology Society at its autumn meeting and, where necessary, updated. To ensure comparability over time only minimal, necessary modifications are made, for example when changes in treatment guidelines or available medical devices occur [5, 6, 11–17]. All changes are made collaboratively, in cooperation with the participating centers, with updates to methods published in subsequent annual reports. Data collection tools and indicator definitions are available on the ANCALAR study homepage: https://iik.i-med.ac.at/ [11].

Each center collects and summarizes their data annually, at the end of the year. During each calendar year, centers are visited or contacted in order to both perform audits and maintain working relationships. Quality control mechanisms have meant thatin Austria 100% of CathLabs submit data to the registry each year. Once 75% of clinics have completed the data entry, the remaining clinics are contacted personally (in-person meeting, telephone call or individual email), so far up to four repeat personal contact attempts have been required. Mass email follow-up, as has also been documented in Switzerland, has limited utility [7].

Using new data from 2017, trends in key cardiac catheterization indicators in Austria, including acute and non-acute PCI use, treatment of ST-elevation myocardial infarction (STEMI), puncture techniques and complications, re-interventions for chronic stent restenosis (REDOs), use of innovative medical devices, electrophysiology and transarterial aortic valve procedures (TAVI) are assessed [11, 18].

Indicators are constructed in line with the published ANCALAR methods, definitions of procedures presented in this paper are available in Supplementary Table 1 [5, 6, 11]. In brief, indicators are constructed using data pooled across all performing clinics and do not exclude cases with missing data in the numerator where denominator data are complete, thus underestimates are likely. To give a more accurate picture of what is happening in Austrian CathLabs, indicators are also constructed using pooled data from subsets of clinics where data are complete.

International comparisons are made with Switzerland and Germany using pooled data from PCI clinics in each respective country, diagnostic coronary angiographies (CA), TAVI and glycoprotein (GP) blockers are compared using both absolute numbers, and crude rates per one million inhabitants, in line with conventional methodology [7–11, 17–21].

## Results

**Fig. 1:**
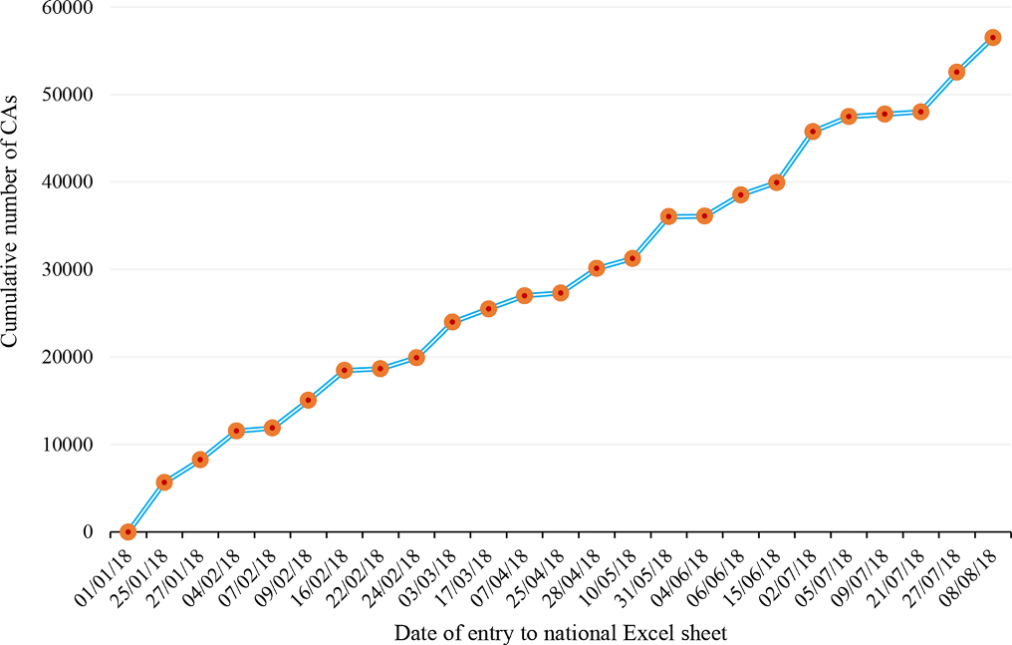
Cumulative numbers of reported diagnostic coronary angiographies (CA) performed in 2017 from the *n* = 34 catheterization laboratories in Austria by date of report during 2018 in the national Excel sheet until 8 August 2018 (*n* = 56,515)

All 34 PCI clinics operating since 2012 in Austria submitted data to the registry for 2017, with a total of 54 CathLab tables between them, in 2017 (Table 1). In 2017, 56,515 CAs were reported (Fig. 1; Table 2).

**Table 1:** Cardiac catheterization Laboratory structure in Austria 2011–2017

Year	2011	2012	2013	2014	2015	2016	2017
Number of centers	36	34	34	34	34	34	34
Number of tables	*49*	50	50	52	53	53	*54*
Number of physicians for diagnostics only ^a^	*243*	261	272	271	291	309	*304*
Number of physicians for diagnostics and PCI^a^	214	222	226	238	250	250	262

Extended questionnaire of the European Society of Cardiology (ESC) [19]

Striking differences are in *italics*

*PCI* Percutaneous Coronary Interventions

^a^The number of active physicians may be overrepresented due to multiple appointments

**Table 2:** Cardiac catheter diagnostics in Austria 2012–2017 across all reporting centers with available data. (Source: Austrian Questionnaire “diagnostics and related procedures”) [11]

Year	2012	2013	2014	2015	2016	2017
*Diagnostic coronary angiography (CA)*	53,064	54,566	56,062	54,853	56,750	56,515
Mortality CA overall (%)	76 (0.14)	61 (0.11)	59 (0.11)	61 (0.11)	59 (0.10)	25 (0.04)
*CA without shock due to infarction*	7969	7769	9467	9210	9453	9263
Mortality CA without shock (%)	29 (0.36)	23 (0.30)	23 (0.24)	20 (0.22)	27 (0.29)	12 (0.13)
*CA with shock due to infarction*	520	434	505	474	429	358
Mortality CA with shock (%)	27 (5.19)	25 (5.76)	28 (5.54)	19 (4.01)	15 (3.50)	11 (3.07)
*Myocardial infarction as complication*	31	28	25	32	32	8
With new Q‑wave	9	9	3	0	0	1
Defined by troponin or CK	24	23	6	32	28	4
*Nonfemoral (radial) approach*	12,055	18,441	20,735	27,673	31,850	*34,627*
*Switch to femoral during procedure*	–	–	–	1500	1702	*1901*
*Local radial artery complications*	–	–	–	–	–	*112*
*Reversible neurological complications*	33	41	37	*48*	37	44
*Irreversible neurological complications*	3	*13*	9	6	10	6
*Vascular peripheral complication*	277	309	264	223	192	*113*
With surgery or transfusion	56	41	49	42	28	*25*
With local injection of thrombin	77	115	105	75	59	*34*
*Adverse reactions to contrast media*	70	70	86	204	201	N.A.
*Left ventricular angiography*	18,163	18,572	11,834	12,628	11,646	*10,941*
*Right heart catheterization*	3142	3288	3515	3401	3489	3368

Striking differences in *italics*

*CK* Creatine (Phospho)Kinase

“–” data not available

### International context

**Fig. 2:**
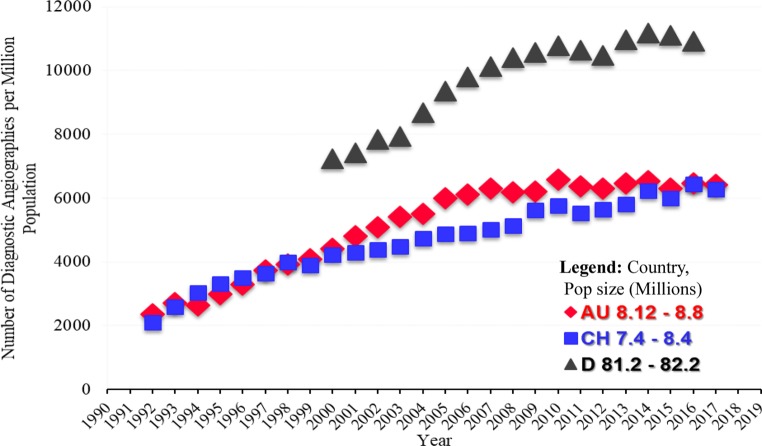
Number of diagnostic coronary angiographies per million inhabitants in Austria (AU), Switzerland (CH) and Germany (D) during the years until 2017 and Germany until 2016 [1–11]. (Source: [18, pp 14])

**Fig. 3:**
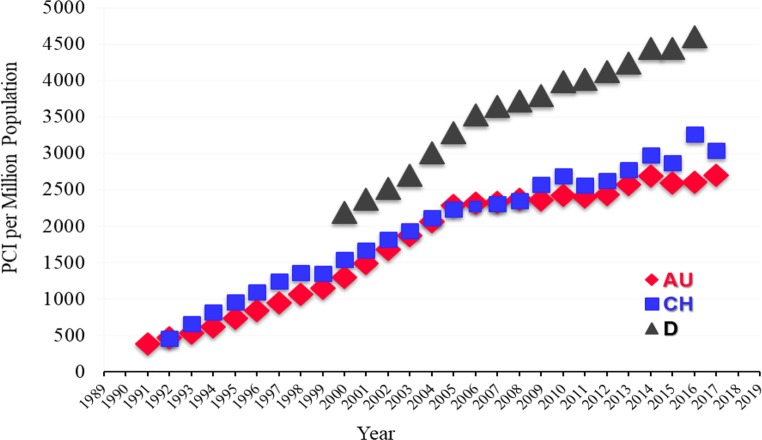
Number of percutaneous coronary interventions (PCI on the y‑axis) per million inhabitants (EW) in Austria (AU), Switzerland (CH) and Germany (D) during the years until 2017, and Germany (D) until 2016 [1–12]. (Source: [18, pp 14])

**Fig. 4:**
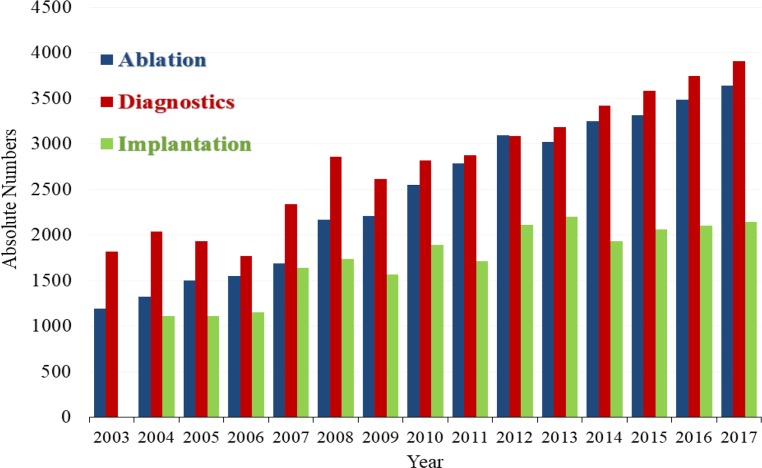
Number of diagnostic electrophysiology, electrophysiological ablations and device implantations in Austrian Cardiac Catherization Laboratories 2003–2017. (Source: [18, pp 23])

In Austria and Switzerland, the absolute numbers of CA are comparable and varied between 2016 and 2017 whilst Germany has consistently higher rates of CA. The PCI/CA ratio increased, with 40.2 of all CAs resulting in PCI in 2016 whilst 42.1% resulted in PCI in 2017 in Austria, comparable with Germany and lower than Switzerland (Table 2; Figs. 2, 3 and 4).

**Fig. 5:**
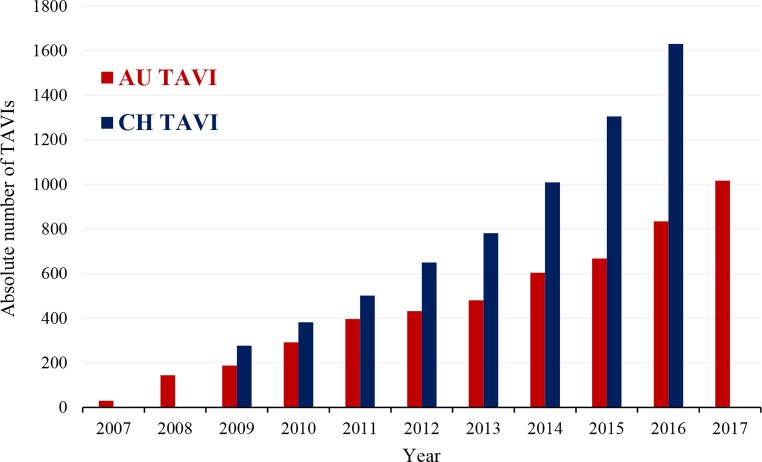
Absolute number of transcatheter aortic valve implantations (TAVI) in Austria during the years 2007–2017 and number of TAVI interventions in Switzerland during the years 2009–2016 [1–9]. (Source: [18, pp 24])

**Fig. 6:**
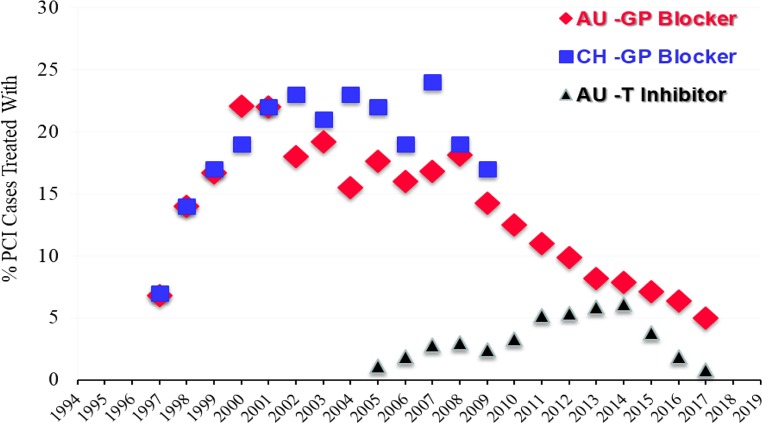
Percentage (%) of cases treated with glycoprotein IIb/IIIa receptor blockers (GP-blocker) per PCI in Austria (AU; 1997–2017) and in Switzerland (CH; 1997–2009) and percentage (%) of cases treated with direct thrombin inhibitors (AU-TI) during PCI in Austria (AU-TI; 2005–2017). [1–13]. (Source: [18, pp 22])

For CA and PCI rates, Austria places just under the top nations in Europe; Austria is in the middle range for TAVI (115 per million population in 2017), with the rate of TAVI per 1 million population increasing year by year (Fig. 5; [17, 21]) Austria began reducing the use of GP blockers years before guidelines reacted to new evidence and at the same time Switzerland stopped counting these cases in their registry (Fig. 6). In contrast, reduction in the use of balloon pumps and catheter thrombectomies in Austria has been protracted (Table 3 and 4; [13–18]).

**Table 3:** Percutaneous coronary interventions (PCI) and related procedures in Austria 2012–2017

Year	2012	2013	2014	2015	2016	2017
*Intracoronary diagnostic device without PCI (cases) e.g. FFR, IVUS, OCT*	–	–	–	1808	*2532*	2148
*PCI (cases) therapeutic interventions*	20,543	21,698	*23,044*	22,538	22,837	23,808
*PCI for acute situation OR ongoing infarction*	7026	7148	7791	8084	8612	*9553 ↑*
PCI for ongoing STEMI	3476	3546	3959	3943	4070	*4581 ↑*
*Bifurcation PCI with large side branch*	989	1081	1175	1454	*1922*	1920
*Multivessel PCI (in one session)*	3231	3094	4309	4300	*4519*	4478
*PCI during diagnostic study (ad hoc)*	17,559	16,085	*18,596*	16,652	16,313	*16,195 ↓*
*Radial/brachial approach (non-femoral) during PCI*	4727	6664	9104	9713	12,551	*13,468 ↑*
*Switch (crossover) to femoral during or before PCI*	–	–	474	479	794	*1017 ↑*
*Local radial artery complication*	–	–	–	–	–	77
*Infarction as complication (by any definition)*	82	78	80	114	*174*	122
*Iatrogenic left main artery dissection*	18	16	24	20	14	27
*Emergency surgery after PCI and/or CA*	19	17	22	19	27	*35 ↑*
*In-hospital death after PCI*	170	185	*243*	205	239	180
*In-hospital death despite emergency surgery post PCI*	1	1	1	1	5	4
*Number of STENT cases:*	18,577	19,995	21,008	20,646	21,257	*22,417 ↑*
Drug eluting stents (cases) (DES)	15,778	17,010	19,451	19,735	20,509	*21,565 ↑*
Drug eluting balloon (DEB) (cases)	723	847	782	937	*1169*	1090
Biodegradable vascular scaffolds (BVS) (aka Biostent)	113	1019	1693	1058	593	*112 ↓*
Left main stents	402	452	473	522	*636*	*636*
Multiple stents (cases)	5360	5668	*8021*	6680	7496	6933
*PCI for in stent restenosis*	687	801	617	814	794	782
PCI due to chronic hyperplasia	329	505	470	559	639	613
PCI due to very late chronic stent thrombosis	82	102	94	103	71	65

Original questionnaire of the European Society of Cardiology (ESC) [19]

cases; *n* pooled analysis

Striking differences in *italics.* Striking changes from 2016 to 2017 are indicated with directional arrows ↑ (increase) ↓ (decrease)

“–” data not available

*FFR* Fractional Flow Reserve, *IVUS* Intravascular Ultrasound, *OCT* Optical Coherence Tomography, *STEMI* ST-Elevation Myocardial Infarction, *CA* Cardioangiography

**Table 4:** Percutaneous CathLab interventions and related procedures in Austria (2012–2017)

Year	2012	2013	2014	2015	2016	2017
Rotablator	312	369	418	373	312	300
Catheter thrombectomy (clot catcher/remover)	1848	1799	1606	1317	1077	*891 ↓*
Intracoronary pressure registration (“fractional flow reserve” (FFR))	2182	2547	2524	3153	3631	3668
FFR decision with adenosine and/or	–	–	–	–	3220	*3164 ↓*
FFR decision without adenosine (= iFR)	–	–	19	64	411	*604 ↑*
PCI for chronic total occlusion (CTO)	637	589	559	790	782	808
Intracoronary ultrasound (IVUS)	816	783	711	670	808	755
Intra-aortic balloon pump during PCI	121	87	82	69	37	*53 ↑*
Other devices (e.g. mechanical circulation support,, protection device) in PCI	53	22	118	102	18	*30 ↑*
Platelet glycoprotein IIb/IIIa antagonist	2025	1775	1815	1597	1467	*1201 ↓*
Direct thrombin inhibitor in PCI	1110	1277	1406	858	439	*198 ↓*
Optical coherence tomography (OCT)	350	570	503	580	*707*	638
Alcohol ablation for septal hypertrophy (PTSMA)	8	14	11	6	13	9

Special techniques, Original questionnaire of the European Society of Cardiology (ESC) [19]

cases; *n* pooled analysis

Striking differences in *italics. *Striking changes from 2016 to 2017 are indicated with directional arrows ↑ (increase) ↓ (decrease)

“–” data not available

*PCI* percutaneous coronary intervention

### Trends in acute and non-acute PCI use in Austria

The number of elective non-acute PCI has plateaued, with the number of cases in 2017 (*n* = 14,255) remaining almost identical to 10 years ago (*n* = 14,254 cases in 2006) (Table 5; [18, 19]); however, the number of patients undergoing non-routine and/or acute PCI (which interrupt daily planned PCI) is increasing year by year (Table 6). In 2017, mortality rates for all acute PCI was 1.64%, although this value is based on the pooled analyzes of all centers, including those with missing data in the numerator, and thus is likely an underestimate of the true PCI mortality rates across Austria.

**Table 5:** Cardiac catheter interventions in Austria 2012–2017

Year	2012	2013	2014	2015	2016	2017
*Nonacute PCI*	13,517	14,550	15,253	14,454	14,225	14,255
Mortality PCI non-acute overall (%)	14 (0.10)	15 (0.10)	25 (0.16)	13 (0.09)	26 (0.18)	23 (0.16)
*Myocardial infarction as complication*	83	78	80	107	174	101
With new Q‑wave	22	11	8	13	15	5
Defined by troponin or CK	58	66	55	79	132	93
*Nonfemoral (radial) approach*	3084	4260	5834	5817	5580	*6868*
*Switch to femoral during procedure*	–	–	–	256	366	*551*
*Local radial artery complications*	–	–	–	–	–	33
*Reversible neurologic complications*	19	14	17	7	11	24
*Irreversible neurologic complications*	4	4	2	1	1	6
*Vascular peripheral complication*	110	123	105	95	*225*	108
With surgery or transfusion	17	32	18	15	*25*	23
With local injection of thrombin	24	32	25	23	*55*	31
*Adverse reactions to contrast media*	27	29	30	24	30	–

Austrian Questionnaire “Non-acute percutaneous coronary interventions PCI” [11]

cases; *n* pooled analysis

Striking differences in *italics*

“–” data not available

*PCI* percutaneous coronary intervention, *CK* Creatine (Phospho)Kinase

**Table 6:** Cardiac catheter interventions in Austria 2012–2017

Year	2012	2013	2014	2015	2016	2017
*Acute PCI (interrupts routine program)* *(intention to treat with PCI)*	7026	7148	7791	8084	8612	9553
Mortality acute overall (%)	156 (2.22)	170 (2.38)	218 (2.80)	192 (2.38)	213 (2.47)	157 (1.64)
*PCI acute without shock*	6537	6754	7316	7648	7648	7867
Mortality PCI without shock (%)	51 (0.78)	68 (1.01)	70 (0.96)	81 (1.06)	78 (1.02)	56 (0.71)
*PCI acute with shock*	489	*394*	*475*	*436*	*467*	*318*
Mortality PCI with shock (%)	96 (19.63)	102 (25.89)	148 (31.16)	111 (25.46)	135 (28.91)	101 (31.76)
*Nonfemoral (radial) approach*	*1319*	*1912*	*2389*	*3004*	*3567*	*3937*
*Switch to femoral during procedure*	–	–	–	144	186	145
*Local radial artery complications*	–	–	–	–	–	29
*Reversible neurologic complications*	10	7	6	4	5	5
*Irreversible neurologic complications*	2	1	1	3	3	2
*Vascular peripheral complication*	90	67	62	34	75	62
With surgery or transfusion	19	17	10	9	12	9
With local injection of thrombin	25	13	7	7	13	18

Austrian Questionnaire *“Acute percutaneous coronary interventions”* *=PCI* in suspected myocardial infarction

cases; *n* pooled analysis

Striking differences in *italics*

“–” Data Not Available

*PCI* percutaneous coronary intervention, *Acute PCI* PCI in patients that interrupt routine program

An increase of complex and acute interventions is evidenced by the increase in STEMI-PCI (Table 3 and 6) to 20.0% of all PCI (in reporting centers) in 2017 (Supplementary Table 2). The number of ad hoc multivessel PCI increased to 20.8% of all PCI in 2017 (Table 3). There is also an increase of PCI in bifurcation of large side branches from 6.7% (2012) to 12.4% (2017) and for left main stents from 2.0% (2011) to 3.3% (2017, Table 6).

Currently 21 centers fulfil the criterion of more than 36 STEMI PCI cases per year, down from a peak of 24 in previous years [20]. PCI for ongoing STEMIs have increased 32% since 2012, emergency surgery after PCI also increased, with some fluctuations, although n’s are small so this result should be interpreted with caution (Table 3). Mortality due to emergency surgery post PCI has more than doubled since 2012 to 11.4% in 2017 (Table 3), although again n’s are small (4 deaths in 35 emergency surgeries) and the definition of emergency surgery has become broader.

The incidence of major bleeding relative to all bleeding complications is declining, especially in acute PCI (from 34.0% in 2010 to 15.8% in 2017) (Supplementary Table 2). Use of glycoprotein IIb/IIIa (5.0%) or thrombin inhibitors (TI, 0.83%) is now extremely rare (Table 4, Fig. 6).

Reinterventions for chronic stent restenosis (REDOs) remain constant at 4.4% of PCI in reporting centers (in 2017 *n* = 782, in 2010: 4.6%, Supplementary Table 2); however, the proportion of very late stent thrombosis as the cause of the reintervention is decreasing, at 9.6% of all REDO’s in 2017 (2016: 11.0%, 2015: 15.4%) (Supplementary Table 2).

### Trends in puncture techniques

**Fig. 7:**
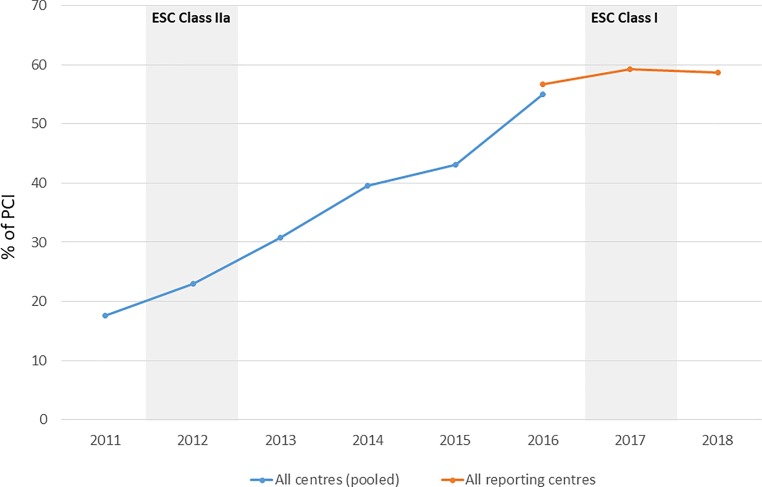
Percentage of PCIs using Radial Access in Austria, 2011–2018

Non-femoral (mostly radial) puncture techniques (Table 2, 3, 5 and 6) in diagnostic CA increased in absolute terms from *n* = 18,441 (2013) to *n* = 34,627 (2017) (Table 2). During diagnostic CA, 6.4% required a switch from radial to femoral (Table 2), with 5.2% of those acute radial cases requiring a switch from radial to femoral during the procedure. Since 2016 there has been a plateau in the use of radial approach (Fig. 7). The number of ad hoc PCIs during diagnostic CA continues to decline (84.4% in 2015 to 75.0% in 2017).

Complications due to radial puncture techniques (Table 2, 3, 5 and 6) were first documented in 2017 [22]. Predictors of radial artery occlusions (RAO) are published by individual centers [22].

### Use of new intracoronary interventional devices

The time of new devices and techniques (innovations) within CathLabs seems few and far between today [23, 24]. For example, use of the drug eluting balloon (DEB), is now declining (Table 4). Declining use of biodegradable vascular scaffolds (BVS) accelerated since 2014. A similar reduction can be seen with catheter thrombectomies (*n* = 891) and intra-aortic balloon pumps (*n* = 53) (Table 4). Left atrial appendage closures (LAA closures), showed a slight renaissance in Austria in 2017 (*n* = 76) (Table 7).

**Table 7:** Percutaneous CathLab interventions and related procedures in Austria (2012–2017)

Year	2012	2013	2014	2015	2016	2017
*Renal, iliac or leg artery intervention in cathlab*	559	475	551	593	*816*	706
*Carotid artery intervention in cathlab*	70	55	52	56	65	*49↓*
*Mitral valvuloplasty*	*42*	–	–	–	–	–
*MitraClip implantation*	51	62	89	91	123	*139 ↑*
*Transcatheter aortic valve implantation (TAVI)*	432	480	604	668	834	*1016 ↑*
Transapical valve (reporting incomplete)	29	35	26	55	46	*133 ↑*
Transarterial valve	403	445	578	613	788	*881 ↑*
*PFO/ASD/PDA closure by catheter*	193	191	218	217	218	198
*Renal denervation (PRD* *=RND)*	151	144	58	29	*14*	–
*Other valve interventions*	–	–	–	–	13	15
*Left atrial appendix (LAA) closure*	–	–	–	–	57	*76 ↑*

Austrian questionnaire *“Non-coronary or non-cardiac interventions”*

(cases; *n* =; pooled analysis).

Striking differences in *italics. *Striking changes from 2016 to 2017 are indicated with directional arrows ↑ (increase) ↓ (decrease)

*PFO* Persisting Foramen Ovale, *ASD* Atrial Septal Defect, *PDA* Persisting Ductus Arteriosus, *PRD* Percutaneous Renal Denervation

“–” or“ Data Not Available

### Extracoronary interventions

The number of procedures on peripheral vessels, e.g. kidneys and legs remained constant, while the number of carotid procedures within the cardiac catheterization laboratories has decreased (Table 7).

Electrophysiology continued to increase in 2017 in all 21 performing centers (Fig. 4). Electrophysiological ablations (*n* = 3640, total) are well established and increasing, of which *n* = 1514 were for atrial fibrillation (AF) and *n* = 396 for ventricular arrhythmia (VT). Of the *n* = 2143 pacemaker implantations within the CathLabs *n* = 157 were “leadless pacemakers”, a real innovation pioneered in 2014 at an Austrian center, now spreading worldwide (Table 8).

**Table 8:** Percutaneous CathLab interventions and related procedures in Austria (2012–2017)

Year	2012	2013	2014	2015	2016	2017
*Myocardial biopsies*	180	226	292	303	340	*356 ↑*
*Diagnostic electrophysiology*	3087	3185	3417	3584	3742	*3906 ↑*
*Electrophysiological ablations*	3098	3019	3254	3313	3482	*3640 ↑*
Ablation in atrial fibrillation (reported since 2013)	–	142^a^	1162	1238	1285	*1514 ↑*
Ablation in ventricular rhythm disorders (reported since 2013)	–	4^a^	230	249	369	*396 ↑*
*DEVICE implantations (pacemakers)*	2109	2198	1932	2061	2102	2143
*Leadless pacemaker*	–	4^b^	32	64	84	*157 ↑*

Austrian questionnaire *“Diagnostics and Electrophysiology”*

cases; *n* pooled analysis

Striking differences in *italics, *Striking changes from 2016 to 2017 are indicated with directional arrows ↑ (increase) ↓ (decrease)

^a^incomplete response

^b^worldwide pioneer

“–” Data Not Available

In all 10 performing centers, increases are found in percutaneous valve implantations or valve replacements, e.g. TAVI/TAVR in 2017 (*n* = 1016), as well as in the MitraClip (*n* = 139) (Fig. 5; Table 7).

A visible phenomenon in 2017 are *n* = 2148 cases with intracoronary (IC) devices (Table 3) but without following therapeutic intervention (11.9% of PCI during 2017, Supplementary Table 2). This results in a rate of 42.4% (2148/5061, Table 4) of cases with IC devices (any) without following therapeutic intervention, such as pressure wire with or without adenosine (FFR; *n* = 3668), IC ultrasound (IVUS; *n* = 755), or optical coherence tomography (OCT; *n* = 638) in reporting centers in 2017. In 2016 the percentage was higher, at 49.2% (2532/5146; Table 4).

### Data quality

The methods of ANCALAR have meant that data for benchmark parameters have been reported by 100% of clinics in each year the data were requested, generating a rich database. For a few specific parameters, particularly indicators of negative outcomes such as severe bleeding during CA or PCI, not all clinics report these data which could lead to underreporting if these outcomes are occurring but are not being reported in the registry. A description of missing data is available in Supplementary Table 2, which notes the exact number of clinics (out of the 34 possible) from which only complete data were pooled to calculate the respective indicator. ANCALAR provides the most comprehensive data concerning cardiac catheterization in Austria today, across all PCI capable health facilities operating in the country.

## Discussion

Austria currently ranks alongside the top countries in Europe in respect to CA and PCI use. As with other countries, complex and acute interventions are increasing year by year in Austria. STEMI-PCI is increasing year by year and now accounts for one fifth of all PCIs, this current trajectory will present logistical challenges given the need for complex cases to be assigned to experienced centers [24, 25].

With respect to international guidelines, Austria provides some interesting insights—guidelines are often slower in their reaction to new evidence than the daily practice of cardiologists. The use of the radial approach in Austria reflects this: over 50% of PCIs were conducted using TRA prior to the ESC classifying the evidence in support of the procedure as class I, in 2016 (Fig. 7); however, since 2016 TRA use has plateaued in Austria as cardiologists react to new evidence that the relative clinical benefits of TRA are less than previously thought, in spite of the current guidelines [23].

Registry data, by its nature, has strengths and weaknesses. ANCALAR has been collecting data on real world cardiology practice in Austria for over 30 years, enabling benchmarking and international comparisons. Personal communication with leading physicians in cathlabs across Austria has meant that year on year every center practicing interventional cardiology in Austria has submitted data to the ANCALAR. ANCALARs methods are transparent and standardized, with onsite audits, cross-validation of data where needed, and centralized data processing, ensuring high quality data that is comparable over time. Throughout each calendar year, leading physicians in all cathlabs offer feedback on ANCALAR, with annual meetings enabling personal discussion between cardiologists about adaptations to indicators and introduction of new indicators. ANCALAR is a valuable resource to cardiologists within both Austria and internationally, its integrity strengthened by its continued financial independence of any person or institution.

As expected with registry data, qualitative issues in definition and reporting make statistical analysis of mortality (Table 2, 3, 4 and 5) increasingly complex*. *For example, the classification of PCI in cardiogenic shock (ICD10: R57.0) leaves a lot of room for manoeuvre. Additionally, the decline of ad hoc PCIs in Austria may well be actually due to the discharge of a patient after radial diagnostics who are considered a “new” admission when a femoral instead of radial puncture for PCI is performed on a separate date. With respect to re-punctures, there are questions about whether switch to femoral access during PCI is also classified as re-punctures or not, leading to potential underreporting due to these qualitative definition issues.

Indeed, underreporting remains a key issue in registry data, not solely due to definitional issues. Of particular note is the potential underreporting and thus subsequent underestimation of mortality rates. It follows that it is is reasonable to expect that the low mortality rate for all acute PCI of 1.64% in 2017 is likely an underestimate due to underreporting and missing data. Many centers may only report mortality for acute PCI if deaths occur “on the cathlab table”, which could also lead to the underestimation of mortality. Additionally, PCI complications are underreported; however, some centers in Austria as well as in Switzerland independently publish their complication rates [7, 18].

Registry data cannot provide answers to causal questions. For example, the link between decreasing peripheral vascular complications and decreasing application of GPI and TI.

Registries are necessarily limited in the amount and type of data they collect. The impact of periprocedural myocardial infarction (MI) is important (Table 2, 3 and 5), yet this area remains underdocumented in the ANCALAR [26]. Additionally, the distinction between restenosis due to chronic hyperplasia or late/very late stent thrombosis is not easily discernible from registry data, particularly given the data may not necessarily be recorded by the interventional physician [25, 27]; however, registry data are the key to highlight current trends in daily practice and provide evidence of the effects of changing practice. For example, there appears to be a decline in PCI for restenosis due to late stent thrombosis in Austria. Maybe the application of dual antiplatelet therapy (DAPT), even in all-comers, is now proving effective [28]. No restenosis is few and far between today [29].

Where feasible, specialist sub-registries are required to supplement registry data. For example, in Austria data on silent closures of radial arteries, higher technical and x‑ray exposure and differential learning curves in radial puncture techniques are available in the special Austrian registry, (http://ptca.i-med.ac.at), which observes STEMI patients [30].

Policy and practice are influenced by cardiac registries. Guidelines can be slow to react to emerging evidence and changes in real world practice. Registries such as ANCALAR can both influence the construction of guidelines and enable cardiologists to understand the “sinn und unsinn” (sense and nonsense) of current guidelines. Moreover, registries such as ANCALAR hold a mirror up to all stakeholders in the world of cardiac intervention, from authorities to cardiologists making everyone more alert to changes in everyday practice. For example, during the autumn conference of the ÖKG working group, which took place on 1 December 2017, in response to new ANCALAR data it was decided that every physician in Austria performing acute PCI should master both the radial and femoral techniques. Indeed, sometimes in interventional cardiology, registries such as ANCALAR are the only and/or most up to date benchmark.

## Conclusion

The most recent results from ANCALAR highlight that interventional cardiology in Austria is, in the main, in line with the top countries in Europe. However, some Austrian idiosyncrasies in response to new evidence and guidelines exist. Often, Austria reacts very early to new evidence and guidelines, as seen by trends in GPIIb/IIIa, radial access and direct thrombin inhibitors. Indeed Austria remains hesitant in adopting new devices, particularly those with niche applications such as aspiration thrombectomy, and avoids “hypes”, such as biostents. Austria is often both ahead of the curve, adapting daily practice before new guidelines are released, whilst simultaneously proceeding with caution, particularly with respect to new devices.

The dynamic nature of cardiac catheterization and increasing number of complex cases has implications for cardiac registries, including ANCALAR. Quantitative changes in complication and mortality rates may in fact reflect qualitative changes in data reporting resultant of such dynamism, cardiac registers and the interpretation of their data need to continue to adapt in the face of such changes.

In conclusion our registry data show that Austria is another example of the difficulties of real life and science meeting in the world of interventional cardiology; with registry data careful interpretation is needed to identify artefacts and understand real differences in the practice of interventional cardiology [31].

## Caption Electronic Supplementary Material

Austrian National Cardiac Catheterization Laboratory Registry Centres 2017/2018 and Pooled Indicators 2015–2017
