# Supplementary material for: Cardiac catherization in Austria: Results from the Austrian National Cardiac Catheterization Laboratory Registry (ANCALAR) 2012–2018
Source: Wien Klin Wochenschr. 2020 Jan 29;132(3):79–89. doi: 10.1007/s00508-019-01599-4 (PMC7035228; doi:10.1007/s00508-019-01599-4)
Supplement: Supplementary file 1 — Austrian National Cardiac Catheterization Laboratory Registry Centres 2017/2018 and Pooled Indicators 2015–2017 [file 508_2019_1599_MOESM1_ESM.docx]

**Supplementary Table 1: Definitions**

| Elective non-acute PCI | Non emergent scheduled PCIs |
| --- | --- |
| Non-routine PCI | Interrupting planned daily schedule |
| Acute PCI | Emergent PCI |
| ad hoc multivessel PCI | Therapeutic intervention performed within the same procedure |
| Bifurcation of large side branches | significant stenosis occurring at or adjacent to division of a coronary artery |
| Left main stents | Stent in the left main coronary artery (LMCA) |
| STEMI PCI | PCI for patient with ST-elevation myocardial infarction (STEMI) |
| Emergency surgery after PCI | Surgery occurring during or immediately after PCI surgery in order to rescue the patient |
| Major bleedings | Peripheral bleeding leading to transfusion or surgery |
| All bleeding complications | Definition varies across centres, includes both major and non-major bleedings |
| Very late stent thrombosis | thrombosis occurring 1 year after stent implantation |
| Non-femoral (mostly radial) puncture techniques | Non-femoral access site (not necessarily radial) |
| Radial puncture | Puncture of radial artery |
| Re-punctures | Re puncture of any artery due to failed approach during the procedure |
| Catheter-door-to-balloon time | Time from admission to balloon |
| Radial Artery Occlusions | Occlusion of radial artery as complication of transradial approach |
| Innovative interventions | Interventions that have not been performed the year before |
| Extracoronary intervention innovations | Not concerning the coronary artery |
| Intracoronary (IC) devices without following therapeutic intervention | IVUS, OCT, pressure wire for diagnostic purposes only, not used in combination with PCI |

**Supplementary Table 2: Indicators (relative percentage %) constructed on Pooled Data from all Respective Reporting Austrian CathLabs Only (n)** 2015-2017 [5-7]

| Year | 2015 | 2016 | 2017 |
| --- | --- | --- | --- |
| Intracoronary diagnostics but without therapy | 13.1 (22) | 12.8 (29) | **11.9** (27) **↓** |
| PCI acute | - | 37.7 (34) | **40.1** (34) ↑ |
| More than 36 STEMI per centre and year | - | (23) | (21) |
| STEMI / PCI | 17.20 (33) | 18.4 (33) | **20.0** (33) ↑ |
| Diagnostic angiography with radial puncture | - | 56.1 (34) | **62.02** (33) ↑ |
| PCI all with radial puncture | - | 56.7 (33) | **59.3** (32) ↑ |
| PCI acute with radial puncture | - | 60.4 (28) | **69.1** (25) ↑ |
| Switch to femoral in diagnostic angiography with radial puncture | 7.8 | 7.0 (24) | **6.4** (27) **↓** |
| Switch to femoral in PCI with radial puncture | - | 9.3 (22) | **8.3** (26) **↓** |
| Switch to femoral in PCI acute with radial puncture | - | 8.1 (20) | **5.2** (20) **↓** |
| Local complication in diagnostic angiography with radial puncture (new question 2017) | - | - | 0.55 (22) |
| Local complication in PCI with radial puncture (new question 2017) | - | - | 0.9 (21) |
| Local complication in PCI acute with radial puncture (new question 2017) | - | - | 1.1 (19) |
| PCI ad hoc during diagnostic angiography | - | 77.4 (31) | **75.0** (31) **↓** |
| PCI in bifurcation vessel | - | 11.4 (26) | **12.4** (23) ↑ |
| Left main stent | - | 3.2 (30) | 3.3 (28) |
| Multivessel PCI in one session | 19.1 (34) | 19.9 (33) | **20.8** (30) ↑ |
| Re-stenosis “REDO” in reporting Centres (% (n) of PCI) | 4.7 | 3.7 | 4.4 (29) |
| REDO due to very late chronic thrombus (% (n) of REDO PCI) | 15.4 | 11.0 (26) | **9.6** (24) **↓** |
| Severe bleeding per bleeding in diagnostic angiography | 20.5 | 21.5 | **23.4** (13) ↑ |
| Severe bleeding per bleeding in PCI elective | 18.5 | 13 | 21.9 (13) |
| Severe bleeding per bleeding in PCI acute | 64 (23) | 18.5 (23) | **15.8** (23) **↓** |
| Mortality due to cardiogenic shock PCI in reporting centres with plausible data | - | - | 34.7 (20) |
| Myocardial infarction post PCI in all reporting centres | - | 1.1 (28) | 0.73 (26) |
| Myocardial Infarction post PCI in reporting centres with plausible data | - | 1.07 (21) | 1.1 (22) |

^% = Percentage within the CathLabs providing data^

^(n = Number of CathLabs providing data, total CathLabs total n= 34)^

**Addendum 1 Austrian Centres for Cardiac Catheterization** 2017/2018 and names of representatives and year of recent visits

| 1) | **Klagenfurt:** Klinikum, Innere Medizin und Kardiologie | *) 2004-2005 |
| --- | --- | --- |
|  | *EOÄ Dr. med. Kornelia Laubreiter, ab 2017: Prim. Priv.-Doz.. Dr. Hannes Alber* |  |
| 2) | **Wien:** Universitätsklinik, Kardiologie, Innere Medizin II | *) 2013 |
|  | *Univ.-Prof.Dr. Bernhard Frey* |  |
| 3) | **Linz:** Krankenhaus der Elisabethinen, Innere Medizin | *) 2006 |
|  | *Prim. Mag. Dr. Josef Aichinger* |  |
| 4) | **Graz:** Universitätsklinikum - LKH, Kardiologie, Innere Medizin | *) 2004 – 2005 + 2013 |
|  | *leitende Assistentin Frau Susanne Knopper, Dr. Helmut Brussee* |  |
| 5) | **Salzburg:** Invasive Kardiologie Prof. Dr. Heyer | *) 2004 - 2005 |
|  | *Prof. Dr. Günter Heyer* |  |
| 6) | **Wien:** Krankenhaus Hietzing (Rosenhügel), 4.Med.Abteilung mit Kardiologie | *) 2010 |
|  | *Prim. Univ. Prof. Dr. Georg Delle Karth, OA Dr. Thomas Publig* |  |
| 7) | **Bad Schallerbach:** Rehabilitationszentrum Austria | *) 2006; 2015 |
|  | *bis 2017: Prim. Dr. Günter Helmreich, ab 2017: Prim. Dr. Günther Stowasser* |  |
| 8) | **Graz:** LKH Graz Süd-West, Kardiologie, Innere Medizin | *) 2004 - 2005 + 2009 |
|  | *Priv.Doz.Dr.H.W.Schuchlenz* |  |
| 9) | **Linz:** Kepler Universitätsklinikum, Med Campus III, früher AKH | *)2004 - 2005 |
|  | *Prim. Priv.-Doz. Dr. Clemens Steinwender, OA Dr. Michael Grund* |  |
| 10) | **Villach:**  LKH, Medizinische Abteilung | *) 2004 - 2005 |
|  | *Fr. OÄ Dr. Anna Rab* |  |
| 11) | **Wien:** Krankenanstalt Rudolfstiftung, Innere Medizin | *) 2004 - 2005 |
|  | Prim. Univ. Prof. Dr. Franz Weidinger, OA Dr. Michael Derntl |  |
| 12) | **Feldkirch:** Landeskrankenhaus, Interventionelle Kardiologie | *) 2004 - 2005 |
|  | *Prim Priv. Doz.Dr. Matthias Frick* |  |
| 13) | **Wien:** Hanusch-Krankenhaus, *II.Med. Abteilung* | *) 2011 |
|  | *Primarius Dr. Johann SIPÖTZ, OA Dr. Michal Winkler, ab 2017*  *OA Dr.Thomas Chatsakos* |  |
| 14) | **Wien:** Privatklinik Josefstadt, Confraternität, ITC Herzkatheterlabor | *) 2012 |
|  | *Frau Bohantsch c/o. OA Dr. Gerhard Bonner* |  |
| 15) | **Schwarzach/St.Veit:** Kardinal Schwarzenberg´sches Krankenhaus,  Interdisziplinäres Gefäßzentrum  *Direktor Dr. Hubert Wallner* | *) 2004 – 2005 + 2014 |
| 16) | **Salzburg:** Landeskrankenhaus, Universitätsklinikum, Innere Medizin | *) 2004 - 2005 + 2006 |
|  | *OA Dr. Wilfried Wintersteller, Primaria Prof. Dr. Uta C. Hoppe* |  |
| 17) | **Bruck an der Mur:** LKH, Hochsteiermark, Abteilung f.Innere Medizin | *) 2008 |
|  | *Prim. Univ. Doz. Dr. Gerald Zenker, OA Dr. Klaus Kaspar* |  |
| 18) | **Wien:** Wilhelminenspital, Innere Medizin und Kardiologie | *) 2004 - 2005 |
|  | *Prim. Univ. Prof. Dr. Kurt Huber, Doz. Dr. Alexander Geppert* |  |
| 19) | **Graz:** Privatklinik Graz Ragnitz, Institut für Interventionelle  Kardiologie und Gefäßmedizin  *Prim. Dr. Ronald Hödl* | Opened in 2016 |
| 20) | **Eisenstadt:** Krankenhaus der Barmherzigen Brüder, Innere Medizin | *) 2004 - 2005 |
|  | *Prim. Priv.-Doz. Dr. Rudolf Maximilian BERGER, OA Dr. Maximilian Juhasz* |  |
| 21) | **Wels:** Klinikum Wels-Grieskirchen, Abteilung für Innere Medizin II mit Kardiologie und Intensivmedizin  *bis 2017: Prim. Univ. Prof. Dr. Bernd Eber, OA Dr. Edwin Maurer,* | *)2004 - 2005 + 2012 |
|  | *ab 2017 Prim. Univ. Prof. Dr. Ronald Binder, Frau Martina Graf* |  |
| 22) | **Krems:** Universitätsklinikum Krems, Innere Medizin | *) 2008 |
|  | *OA Dr. Miklos ROHLA, Prim. Univ. Prof. Dr. Thomas Neunteufl OA Dr. Mario Zangrando ab 2018* |  |
| 23) | **St.Pölten:** Universitätsklinikum, 3. Medizinische Abteilung | *) 2008 |
|  | *Prim. Univ. Prof. Dr. Harald Mayr, OA Dr. Paul Vock* |  |
| 24) | **Innsbruck:** Universitätsklinik, Innere Medizin III, Kardiologie | *) 2015 to 2018 |
|  | *Univ.Prof. Dr. Guy Friedrich,* Univ.-Prof. Dr. Günter Weiss |  |
| 25) | **Lienz:** Bezirkskrankenhaus, Interne Abteilung | *) 2009 |
|  | *Prim. Univ. Prof. Dr. Peter Lechleitner, OA Dr.Peter Lukasser* |  |
| 26) | **Wien:** SMZ Ost, Donauspital, 1. Medizinische Abteilung | *) 2008 |
|  | *Prim. Prof. Dr.Thomas Stefenelli, OA Dr. Georg Norman* |  |
| 27) | **Mistelbach:** Landesklinikum Mistelbach-Gänserndorf, Innere Medizin I | *) 2008 |
|  | *Prim. Univ. Doz. Dr. Otto Traindl* |  |
| 28) | **Wiener Neustadt:** Landesklinikum; Innere Medizin - Kardiologie und Nephrologie | *) 2010 |
|  | *Prim. Univ.-Doz.Dr. FX Roithinger, ab 01/2018 Doz. Dr. Martin Haas,*  *zuvor OA Dr. Ch. Rott,* |  |
| 29) | **Wien:** Wiener Privatklinik | *) 2013 |
|  | *leitende Assistentin Frau Krista KEIBLINGER, Univ.-Prof. Dr. Martin Schillinger* |  |
| 30) | **Mödling:** Landesklinikum Baden- Mödling, Innere Medizin mit Kardiologie, *geschlossen am 1.9.2017* | *) 2008 |
| 31) | **Wien:** Rudolfinerhaus, Institut für Invasive Kardiologie | *) 2008 |
|  | *OA Dr. T. Brunner* |  |
| 32) | **Waidhofen/Ybbs:** Landesklinikum, Innere Medizin | *) 2008 + 2009 |
|  | *Prim. Dr. Martin Gattermeier, OA Dr. Gerhard Bonner* |  |
| 33) | **Wien:** SMZ-SÜD / KFJ- Spital, 5.Med.Abt. | *) 2009 |
|  | *Frau. Prim.Doz. Dr. A.Podczeck-Schweighofer, Univ.Prof. Dr.G. Christ* |  |
| 34) | **Klagenfurt:** Maria Hilf, privates Katheterlabor | Opened in 2012 |
|  | *Prim. Dr. Josef Sykora, Dr. Heinz Krappinger* |  |
